# Tick-borne Encephalitis from Eating Goat Cheese in a Mountain Region of Austria

**DOI:** 10.3201/eid1510.090743

**Published:** 2009-10

**Authors:** Heidemarie Holzmann, Stephan W. Aberle, Karin Stiasny, Philipp Werner, Andreas Mischak, Bernhard Zainer, Markus Netzer, Stefan Koppi, Elmar Bechter, Franz X. Heinz

**Affiliations:** Medical University of Vienna, Vienna, Austria (H. Holzmann, S.W. Aberle, K. Stiasny, F.X. Heinz); Regional Hospital, Rankweil, Austria (P. Werner, S. Koppi); Austrian Public Health Authorities, Vorarlberg, Austria (A. Mischak, B. Zainer, M. Netzer, E. Bechter)

**Keywords:** TBE virus, outbreak, oral transmission, high altitude, goat milk, meningo-encephalitis, viruses, changing epidemiology, dispatch

## Abstract

We report transmission of tick-borne encephalitis virus (TBEV) in July 2008 through nonpasteurized goat milk to 6 humans and 4 domestic pigs in an alpine pasture 1,500 m above sea level. This outbreak indicates the emergence of ticks and TBEV at increasing altitudes in central Europe and the efficiency of oral transmission of TBEV.

Tick-borne encephalitis virus (TBEV) is a human pathogenic flavivirus that is endemic to many European countries and to parts of central and eastern Asia (*1*). Even though vaccination can effectively prevent TBE (*2*), >10,000 cases are reported annually for hospitalized persons in areas of Europe and Asia to which TBE is endemic. TBEV occurs in natural foci characterized by ecologic habitats favorable for ticks, especially in wooded areas within the 7°C isotherm (*3*). The major route of virus transmission is tick bites, but TBEV also can be transmitted during consumption of nonpasteurized milk and milk products from infected animals, primarily goats (*3*). Outbreaks resulting from oral virus transmission are rare in central Europe but more common in eastern Europe and the Baltic states (*3*). Our investigation of TBEV transmitted by milk from a goat in an alpine pasture in a mountainous region provides evidence for a changing TBEV epidemiology in central Europe and the expansion of ticks and TBEV to higher regions.

## The Study

We investigated a TBE outbreak, comprising 6 cases, in a mountain region in western Austria in July 2008. The index case occurred in a 43-year-old shepherd who had stayed for 24 days at his alpine pasture (1,564 m above sea level) before he was hospitalized for nonbacterial urethritis and nonspecific influenza-like symptoms (including pain in the lower abdomen and legs), followed by clinical signs of meningitis. TBEV infection was confirmed serologically by ELISA demonstration of specific immunoglobulin (Ig) M and IgG in serum and cerebrospinal fluid. The patient did not remember a tick bite but had eaten self-made cheese prepared from a mixture of nonpasteurized goat milk and cow milk 8–11 days before illness onset; further investigation found 6 additional persons who had eaten the same cheese (Figure). For 5 of them, recent TBEV infection was serologically proven (Table). For 3 of these persons (2 men, 44 and 65 years of age; and 1 woman, 60 years of age), similar to the index patient, a typical biphasic course and symptoms of TBE (nonspecific flu-like symptoms followed by fever, cephalea, meningism, and ataxia after 4–10 days) developed and they were hospitalized. The 2 other persons who had eaten the cheese (female, 37 and 7 years of age) were clinically asymptomatic. The noninfected person had vomited shortly after eating the cheese because of a gastric banding. None of the infected persons had been vaccinated against TBEV.

**Figure:**
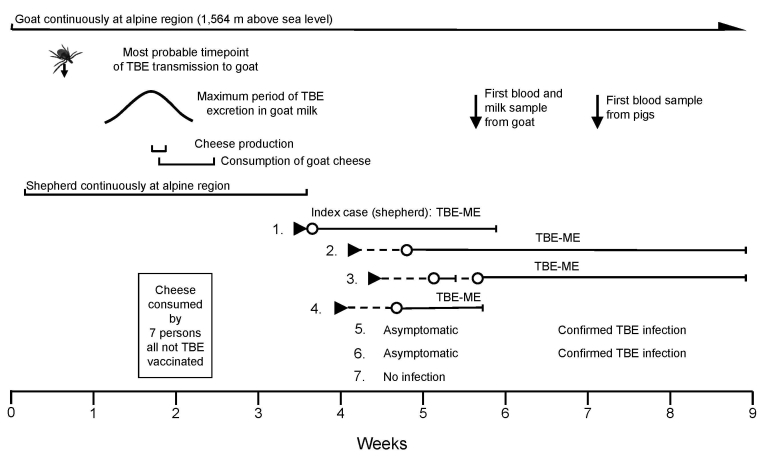
Time course and series of events of a tick-borne encephalitis (TBE) outbreak from cheese made with goat milk. Week 0, transport of goat to high altitude; ►, onset of disease; O—I, hospitalization period; TBEV, tick-borne encephalitis virus; ME, meningoencephalitis.

**Table:** Infection parameters of 7 persons exposed to TBEV by eating nonpasteurized goat cheese, Austria, 2008*

Sex/ age, y	Incubation, d	Symptoms/signs	Diagnosis	Hospitalized, d	Virologic parameters				TBEV infection confirmed
					Material	TBEV ELISA		TBEV NT	
						IgM	IgG		
M/43	11	Fever, cephalea, meningism, aseptic urethritis; CSF: pleocytosis	ME	18	Serum	Pos	Pos	Pos	Yes
					CSF	Bor	Pos		
M/65	10	Fever, cephalea, meningism, vertigo, cerebellar ataxia; CSF: pleocytosis	ME	30	Serum	Pos	Pos	Pos	Yes
					CSF	Bor	Bor		
F/60	14	Fever, cephalea, meningism, vertigo, cerebellar ataxia; CSF: pleocytosis	ME	25	Serum	Pos	Pos	Pos	Yes
					CSF	Pos	Pos		
M/44	9	Fever, cephalea, meningism, vertigo, cerebellar ataxia; CSF: pleocytosis	ME	9	Serum	Pos	Pos	Pos	Yes
					CSF	Pos	Bor		
F/37	NA	None	NA	0	Serum	Pos	Pos	Pos	Yes
F/7	NA	None	NA	0	Serum	Pos	Pos	Pos	Yes
F/45	NA	None	NA	0	Serum	Neg	Neg	Neg	No

*TBEV, tick-borne encephalitis virus; NT, neutralization test; CSF, cerebrospinal fluid; Ig, immunoglobulin; ME, meningioencephalitis; pos, positive; bor, borderline; NA, not applicable; neg, negative.

The cheese was prepared from a mixture of fresh milk from 1 goat and 3 cows and was eaten shortly after production. Detection of TBEV-specific hemagglutination inhibiting (HI) and neutralizing antibodies in the goat’s serum proved infection in the goat; the 3 cows were seronegative for TBEV. At the time of this investigation (1 month after cheese production), TBEV was already undetectable by PCR in serum and milk of the goat. Cheese from the 3 batches produced after the contaminated batch was TBEV negative by PCR. The original cheese was no longer available for testing.

The 4 domestic pigs kept at the alpine pasture and fed with the whey and goat milk, however, were seropositive (TBEV HI- and neutralizing antibodies detected), which indicated TBEV infection, but no clinical signs were observed. Infection with TBEV has been reported in wild boars (*4*,*5*). Serum samples from 105 goats from pastures in the neighborhood also were investigated for TBEV-specific antibodies; all goats were seronegative.

## Conclusions

Our analyses showed that the 6 humans and the 4 pigs were infected through the milk of 1 goat, which had been transported by car from a TBE–nonendemic valley to the alp 12 days before production of the TBEV-contaminated cheese. Experiments have demonstrated that infected domestic animals (i.e., goats, sheep, and cows) can excrete TBEV into milk for ≈3–7 days, beginning as early as the second or third day postinfection (*6**–**9*). In addition, although cheese was produced once or twice each week, only this ≈1-kg batch of cheese transmitted TBEV. Therefore, all the evidence indicates that the goat was infected at the alpine pasture at an altitude of 1,564 m. Indeed, some ticks were collected from cows that had stayed at this altitude during the entire summer. Analyses of these ticks for TBEV by PCR, however, yielded only negative results.

Our findings provide further evidence for the expansion of TBEV-endemic regions to higher altitudes in central Europe. For example, longitudinal studies in the Czech Republic, a country with similar climatic and ecologic conditions to those of Austria, showed a shift in *Ixodes ricinus* ticks and TBEV, from 700 m in 1981–1983 to 1,100 m altitude in 2001–2005 (*10*,*11*). Likewise, Zeman and Benes demonstrated that the maximum altitude at which TBEV is found in the Czech Republic gradually moved upward during 1970–2000, corresponding to the rise in temperature during the same period (*12*). In Scandinavia, a northward extension of the geographic range of *I. ricinus* ticks and TBEV since the mid-1980s has also been recognized (*1*,*13*–*15*). Climatic changes most likely are the major driving forces for the geographic changes in the distribution of TBEV and its main vector, *I. ricinus*, in Europe.

This report also emphasizes the efficiency of oral transmission of TBEV to humans and to pigs. Six of the 7 persons who ate the cheese and all 4 pigs fed residual milk or whey from the same cheese became infected. Given the excellent effectiveness of the TBE vaccine (*2*), vaccination probably could have prevented all 6 human cases.
